# Towards Automated and High-Throughput Quantitative Sizing and Isotopic Analysis of Nanoparticles via Single Particle-ICP-TOF-MS

**DOI:** 10.3390/nano13081322

**Published:** 2023-04-09

**Authors:** Benjamin T. Manard, Veronica C. Bradley, C. Derrick Quarles, Lyndsey Hendriks, Daniel R. Dunlap, Cole R. Hexel, Patrick Sullivan, Hunter B. Andrews

**Affiliations:** 1 Chemical Sciences Division, Oak Ridge National Laboratory, Oak Ridge, TN 37831, USA; 2 Elemental Scientific, Inc., Omaha, NE 68122, USA; 3 TOFWERK AG, 3645 Thun, Switzerland; 4 Radioisotope Science and Technology Division, Oak Ridge National Laboratory, Oak Ridge, TN 37831, USA

**Keywords:** nanoparticles, automated, high throughput, single particle, ICP-TOF-MS, SP-ICP-MS

## Abstract

The work described herein assesses the ability to characterize gold nanoparticles (Au NPs) of 50 and 100 nm, as well as 60 nm silver shelled gold core nanospheres (Au/Ag NPs), for their mass, respective size, and isotopic composition in an automated and unattended fashion. Here, an innovative autosampler was employed to mix and transport the blanks, standards, and samples into a high-efficiency single particle (SP) introduction system for subsequent analysis by inductively coupled plasma–time of flight–mass spectrometry (ICP-TOF-MS). Optimized NP transport efficiency into the ICP-TOF-MS was determined to be >80%. This combination, SP-ICP-TOF-MS, allowed for high-throughput sample analysis. Specifically, 50 total samples (including blanks/standards) were analyzed over 8 h, to provide an accurate characterization of the NPs. This methodology was implemented over the course of 5 days to assess its long-term reproducibility. Impressively, the in-run and day-to-day variation of sample transport is assessed to be 3.54 and 9.52% relative standard deviation (%RSD), respectively. The determination of Au NP size and concentration was of <5% relative difference from the certified values over these time periods. Isotopic characterization of the ^107^Ag/^109^Ag particles (*n* = 132,630) over the course of the measurements was determined to be 1.0788 ± 0.0030 with high accuracy (0.23% relative difference) when compared to the multi-collector–ICP-MS determination.

## 1. Introduction

Dedicated efforts in analytical chemistry are often centered on minimizing sample handling, improving analysis efficiency, and increasing throughout. These attributes have generally been shown to improve the overall data quality while reducing analysts’ time on the instrument. For inorganic mass spectrometry, the automation of sample introduction and sample preparation has become a major target as a way to boost productivity and sample throughput while reducing the time required for sample preparation [1]. These efforts often manifest in the form of multiport and switching valves [2], sample loops [3], vacuum and syringe pumps [4], mixing chambers [5], and flow injection techniques, such as separation or preconcentration columns [1,4,6,7]. Specifically, in the field of nuclear analytical chemistry, Metzger et al. utilized an automated separation system to separate uranium and plutonium from environmental sample swipes. These automated efforts not only improved sample throughput but also lowered method blanks due to an enclosed separation system which minimized sample handling [8]. Xu et al. utilized a novel sample introduction platform to minimize sample volumes of plutonium metal samples by approximately 90% by introducing the sample via syringe drive in conjunction with a sample loop (as opposed to traditional aspiration), while improving the method detection limit (by approximately 50×) [9]. Aside from nuclear analytical chemistry [10,11], other applications emphasizing improved automation and sample delivery have been particularly useful in terms of ICP-MS-based analysis, including speciation [12], stable isotope analysis [13], and clinical studies [14,15].

Single particle (SP) analysis is a unique sample scheme/approach for ICP-MS-based measurements [16,17,18]. Since the emergence of SP analysis, there have been some efforts explored regarding automated high-efficiency sample introduction [19,20] or automated on-line purifications [21] for single particles/single cells. In comparison to a bulk dissolution, which ultimately homogenizes the sample, SP analysis allows for the direct discrimination of particles. This technique has proven its utility covering a wide range of applications and is revolutionizing the way nanomaterials/nanoparticles (NPs) are studied. In short, this measurement needs a highly efficient way to introduce the sample into the ICP, such that one particle enters at a time. Secondly, an ICP is needed with a mass analyzer fast enough to detect such events. Sector field (SF)-based mass spectrometers are valuable when highly sensitive measurement are warranted, but are often hindered when more than one detected isotope is of interest due to magnet settling time [22]. While more recent hardware and software developments now permit for such fast data acquisition [23], the sequential nature of quadrupole- and single-collector SF-ICP-MS, does not allow for simultaneous multi-elemental NP analysis and thereby hinders high-throughput measurements. Indeed, as the sample needs to be repeatedly analyzed for all isotopes of interest and a sufficient number of NPs for reliable statistics, the analysis time is significantly lengthened. Time-of-flight (TOF)-based instruments appear to present the best utility for SP measurements, not only due to their inherently fast acquisition times, but also to their ability to quasi-simultaneously monitor all masses (e.g., ^6^Li–^242^Pu) [24]. This lends itself to be the ideal platform for isotopically characterizing multi-elemental SPs with ultra-fast acquisition times (a multi-collector instrument could isotopically characterize individual particles, but is limited based on acquisition times and mass range) [25].

Regarding sample introduction considerations for SP analysis, one must consider the effective preparation of the particle suspension, how to sample effectively and quickly, and how to introduce the sample into the ICP with high transport efficiency. Presented here are results from an optimized sample introduction method for NP characterization. A focus was placed on efficiently introducing particles into the ICP-MS with high in-sequence repeatability and successful day-to-day reproducibility. This was achieved with an innovative autosampler which incorporates in-run mixing of the samples, syringe-driven sample uptake for the precise aliquoting of samples, and a state-of-the-art nebulizer, spray chamber, and torch/injector for high-efficiency sample transport. This sample introduction methodology was validated with ionic and NP standards to determine NP concentration, size distribution, and isotopic abundance. The ability to reproductively introduce, detect, and characterize NPs, over long sequences (8 h) and multiple days (5 d) is highlighted here and can have an immediate impact across various applications (e.g., environmental, clinical, etc.).

## 2. Experimental

### 2.1. Materials & Reagents

Monodisperse spherical gold nanospheres (Au NPs) of 50 and 100 nm, and silver shelled gold core nanospheres (Au/Ag NPs) (nanoComposix, Fortis Life Sciences, San Diego, CA, USA) were utilized in the presented work. The certificate of analysis (COA) for these particles is summarized and presented in Table 1. For preparation of the NPs presented here, the stock solutions were diluted such that the working concentration was roughly 50,000 particles mL^−1^. Prior to each dilution, the sample was sonicated for 30 s to aid in resuspension. All dilutions were performed with ASTM type I water (18.2 MΩ·cm) generated with a ThermoScientific Barnstead^TM^ GenPure^TM^ xCAD Plus ultrapure water purification system (Waltham, MA, USA). For quantification, single element ionic standards of Au and Ag were prepared from a stock solution (10 µg mL^−1^) from High Purity Standards (HPS, Charleston, SC, USA). Appropriate dilutions were performed in ASTM type I water to final concentrations of 1, 5, and 10 ng mL^−1^. Optima^TM^ grade nitric acid (HNO_3_) from Fisher Scientific (Pittsburg, PA, USA) was utilized to digest the Au/Ag NPs for subsequent Ag isotope verification via multi-collector (MC) ICP-MS.

### 2.2. Multi-Collector–Inductively Coupled Plasma–Mass Spectrometry

For isotopic verification of the 15 nm silver shell of the Au/Ag NPs, a multi-collector–inductively coupled plasma–mass spectrometer (MC-ICP-MS) was employed. Here, the Au/Ag NPs were dissolved in 4 M HNO_3_ at 150 °C for 30 min. The dissolved material was screened on a Thermo Scientific (Bremen, Germany) TQ ICP-MS to ensure complete dissolution of the Ag shells. Subsequent dilutions were made with 2 % (*w*/*w*) HNO_3_ such that a working concentration of 1 ng mL^−1^ could be analyzed by the MC-ICP-MS (Neptune Plus, Thermo Scientific, Bremen, Germany). Isotopes of Ag were analyzed using two 10^11^ Ω resistance amplifiers with ^107^Ag on the axial cup and ^109^Ag on the H2 cup to allow for periodic recalibration of the center mass position during analysis. A quartz spray chamber was used with a 150 µL min^−1^ nebulizer which resulted in 1 V ppb^−1^ Ag sensitivity. The analysis method was set up as single block of 10 cycles with an 8 s integration time. An HPS Ag standard was run, bracketing the samples to correct for instrumental mass bias effects. The isotopic ratio of the samples was corrected by direct comparison to the Ag standard.

### 2.3. Single Particle–Inductively Coupled Plasma–Time of Flight–Mass Spectrometry

**Figure 1:**
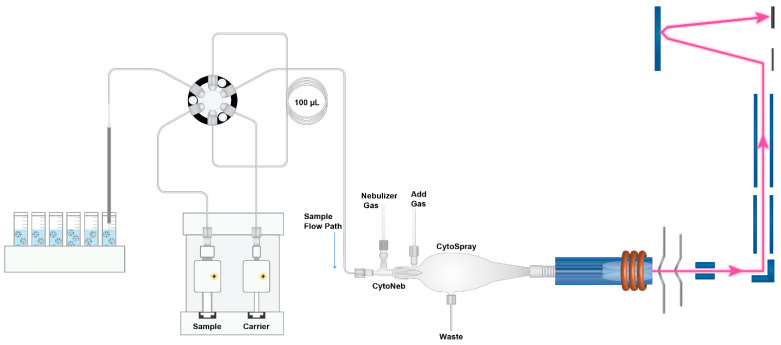
Illustration of the single particle (SP)-ICP-TOF-MS setup.

An Elemental Scientific Inc., (ESI, Omaha, NE, USA) microFAST SC sample introduction system was employed to introduce the NPs to the ICP-TOF-MS. The entirety of this introduction/detection system can be seen in Figure 1, operating conditions can be found in Table 2, and the sample introduction protocol is presented in Table 3. A tapered tipped carbon fiber autosampler probe is directed into the sample in which a mixing step is initiated, to assure NP suspension. Initial studies were performed to determine the optimum mixing protocol. In these studies, multiple repeated measurements were performed with and without mixing. It was found that the sample mixing prior to injection (100 µL) improved the percent relative standard deviation (%RSD) from 12.4 to 5.2 of the number events between samples. This study was performed over a 2 h analysis of 20 samples, each containing approximately 1000 particles. Once mixed, a syringe was employed to transport the sample into the sample loop to deliver a precise volume (100 µL) to the ICP-TOF-MS. Once injected out of the sample loop, the sample is then directed into a CytoNeb50 (ESI) nebulizer housed within a CytoSpray chamber (ESI, SC-CytoC-73) for high-efficiency NP introduction. The aerosolized particles are then transported into a unique one-piece torch/injector (ESI, T20-73, 2 mm) for NP delivery to the ICP. To determine particle transport efficiency, 50 nm Au NPs were injected at a known volume and known particle number concentration (PNC). The injection parameters were optimized such that the particle transport efficiency, calculated via particle frequency, was >80% for the 50 nm Au NPs. This high-efficiency particle transport performance is on par with what has recently been published [26], employing the same introduction components.

Here, an ICP-TOF-MS (icpTOF R, TOFWERK AG, Switzerland) was employed for sample analysis. Uniquely this ICP-MS can quasi-simultaneously detect all isotopes (2–290 Th) at ultra-fast acquisition times (30 µs). Further information regarding this ICP-TOF-MS instrument design can be found elsewhere [24]. This capability allows for multi-element as well as isotopic analysis of single NPs. The tuned mass resolving power and sensitivity of the instrument for ^238^U at the approximate times of measurement was 3100 Δm m^−1^ and 40,000 cps ppb^−1^, respectively. Experiment setup, data acquisition, and data processing were performed using TOFpilot v2.11.5 (TOFWERK AG, Switzerland). Using the dedicated particle module (TOFpilot v2.11.5, TOFWERK AG, Switzerland), the data were thresholded for particle identification by applying the compound Poisson algorithm [27] and subsequently, quantified using liquid standards [28]. Further data processing and data visualization were performed with in-house developed Python scripts.

## 3. Results and Discussion

### 3.1. Reproducibility of Detected Particles

For the characterization of NPs, a sequenced approach was utilized and is presented in Table 3. Briefly, the ASTM Type I water (Blank) was bracketed before and after the sample analysis to monitor for successful rinsing of the tubing and injection loop prior to the analysis of the subsequent sample. Prior to the analysis of the NPs, single element (Au and Ag) ionic standards were measured at varying concentrations such that quantification based on external calibration could be utilized. All blanks and single element standards were measured with a 100 ms integration time for 60 s. These samples also aided in the determination of limits of detection (LOD). All NP samples were analyzed 10× with a 2 ms integration time for 450 s. The entirety of this sequence was completed in ~8 h. It should be noted that all calibration standards and NPs were prepared fresh prior to each daily sequence.

**Figure 2:**
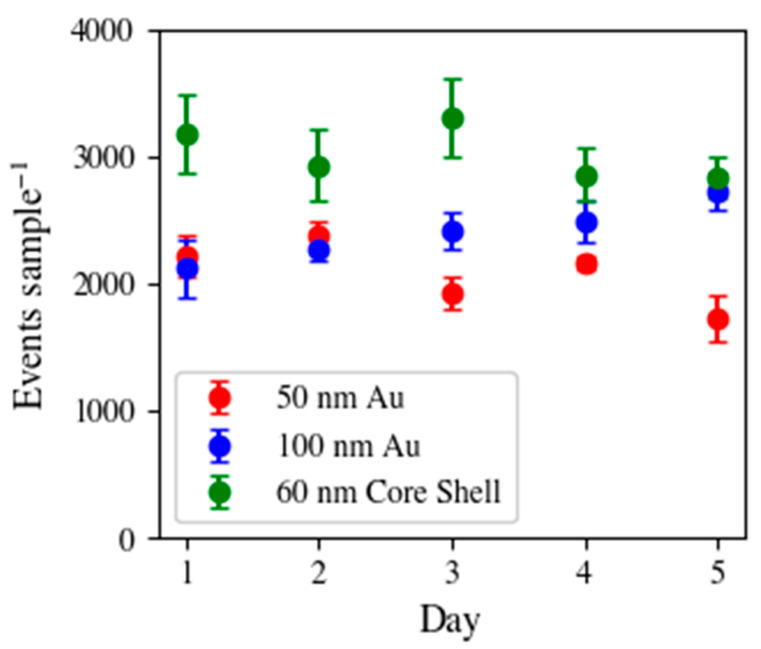
Detection of 50 nm Au (red), 100 nm Au (blue), and 60 nm Au/Ag core-shell (green) NPs presented as the average number of events within the replicate injections [50 nm NPs: *n* = 10, 100 nm and 60 nm Au/Ag NPs: *n* = 11] with its respective standard deviation (2σ) over the course of 5 days.

To determine the reproducibility of precisely injecting the same number of particles in each sample injection, the determined events were compared. For example, in a single injection (100 µL) of 50 nm Au NPs (with the presented dilution factors in Table 1) would yield around 2200 events during the detection time, assuming an 80% transport efficiency. This value is then compared within a single sequence and over the course of days, presented in Figure 2. This provides in-run and day-to-day statistics regarding the reproducibility of the 50, 60, and 100 nm SP introduction into the ICP-TOF-MS. The average %RSD for the in-run precision of detected events of the 50, 60, and 100 nm particles was 3.54 ± 1.05%. For day-to-day precision, the 50, 60, and 100 nm particles were detected at 2092 ± 510, 2412 ± 456, and 3029 ± 419 (2σ), respectively. Ultimately, this provides insight into the reproducibility of the effectiveness of particle introduction. The transport efficiency was calculated daily with an average of 77 ± 6% for the 5 days. As mentioned above, without the automated mixing of the sample, %RSDs (in terms of detected particle events) were seen in the >10% range on a single measurement. It would be expected that this %RSD would greatly increase over the course of longer sequences, such as those presented here. The fact that the average in-run precision during this study was 3.54 ± 1.05% and the day-to-day was 9.52 ± 5.3% demonstrates this introduction approach is a unique and robust way to introduce SP samples into the ICP-MS, which would be greatly beneficial in the arenas of environmental, production, and characterization of NPs.

### 3.2. Mass and Size Characterization of Nanoparticles

**Figure 3:**
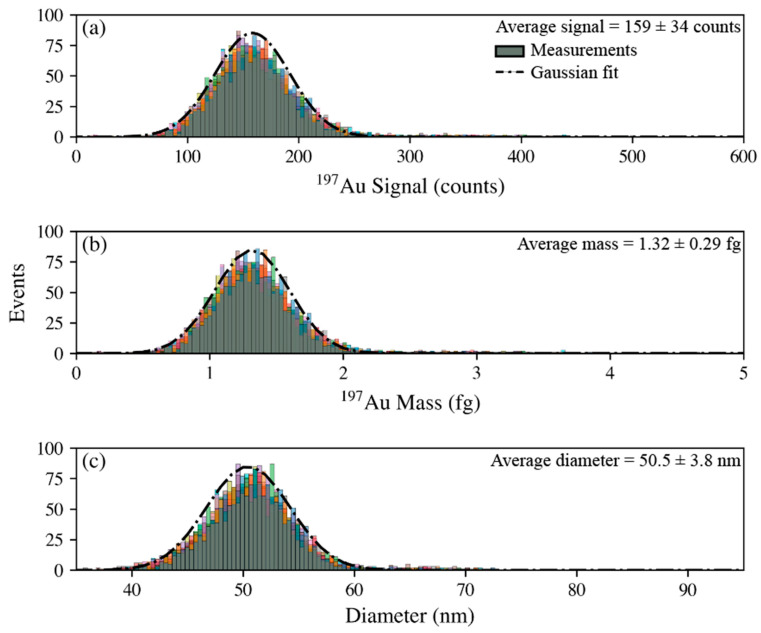
Representative demonstration of particle size calculation for the 50 nm Au NPs (*n* = 10) based on the (**a**) counts, (**b**) mass, and (**c**) calculated size presented as histograms regarding the frequencies of events.

The mass respective size characterization of the 50 nm Au NPs is presented in Figure 3 for a single day. This data clearly demonstrates the effectiveness of accurately determining the mass and size of the NPs. The data were processed in TOFpilot such that the signal (Figure 3a) from the ICP-TOF-MS measurement is converted to mass (Figure 3b) via external calibration based on the method of Pace et al. [29], this mass can then be used to determine NP size with the assumption of a spherical geometry. A set of known information, including elemental distribution and density of respective element (e.g., density of Au is 19.32 g cm^−3^) along with shape of the particles, can be used to calculate the particle diameter. Similar approaches have been previously reported [29,30]. Next, a k-clustering algorithm was applied to identify if multiple gaussian distributions were present in the sample population. The k-clustering was run 10× with differing random seeds for each sample to ensure if multiple distributions were detected that they were repeatedly identified. For the 100 nm Au NPs, a second population with a slightly larger diameter (approximately 106 nm) was identified; for the calculation of the NP diameter to compare with the COA values, only the lower distribution was used. Here, the Au NP sizes were determined to be 50.9 ± 0.30 and 104.9 ± 0.73 nm, over the course of the study, which is in excellent agreement with the certificate value of 51.0 ± 1.9 and 102.2 ± 4.2 nm. The Au/Ag NPs were analyzed with the same procedure, with the addition of the Ag layer thickness calculation. Here, the Au core was calculated to be 31.5 ± 0.32 nm and the Ag thickness was calculated to be 8.76 ± 2.4 nm compared to the certificate values of 30 ± 3 nm and 15 nm, respectively. The difference between the calculated and certificate values of the shell thickness may be caused by Ag layer degradation, or the certificate value may not be very accurate, as it was calculated rather than measured and does not have an associated uncertainty.

**Figure 4:**
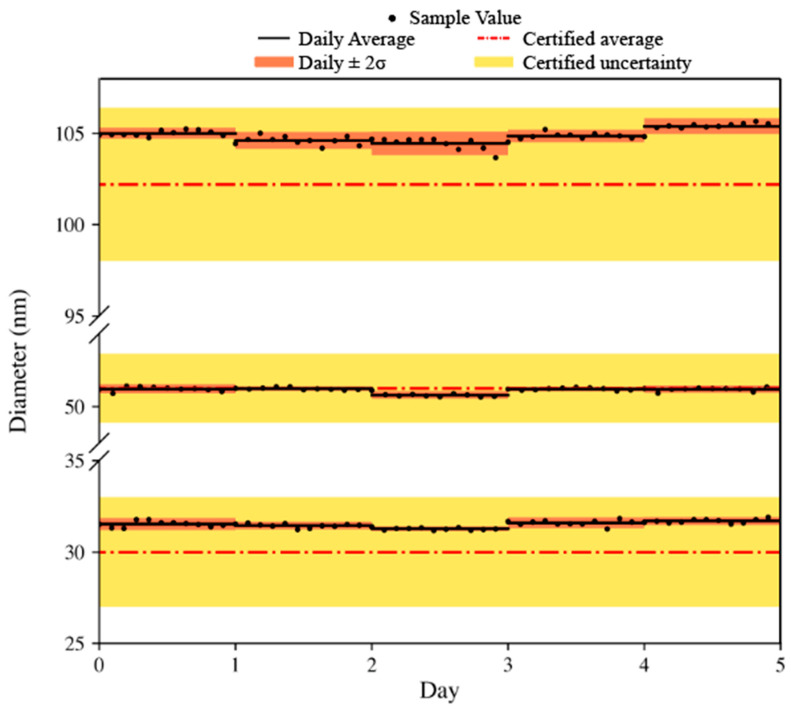
Determination of the Au diameter by SP-ICP-TOF-MS presented for the 50 nm Au, 100 nm Au, and 60 nm Au/Ag NPs presented with the individual sample measurements for each day over the course of 5 days. Each day is presented with its average value (black line) and standard deviation at 2σ (orange shaded box). All data are compared to the certified value (orange dashed line) with its respective standard deviation (yellow shaded box).

The derived particle diameters are presented in Figure 4, showing in-run and day-to-day variation with this characterization. Regarding each sample measurement (e.g., 50, 100 Au NPs and 60 nm Au/Ag NPs), the determined diameter of the Au cores was well within the certificate value with an average diameter (over the course of 5 days) corresponding to a 0.20, 2.6, and 5.0% relative difference, respectively. The in-run precision was assessed as 0.51% RSD on average for all measurements and the day-to-day %RSD being <1.03%.

### 3.3. Isotopic Characterization of Nanoparticles

The isotopic ratio characterization of particles via SP-ICP-MS is of interest to various communities, including environmental [31], nuclear [32], and biological [25]. Typically, this measurement would require the ability to simultaneously measure multiple isotopes of interest, such that single particle discrimination can be achieved. To date, and the authors’ knowledge, SP-ICP-MS of suspension for isotopic determination has only been primarily investigated with either a TOF [25,31] or SF-MC [25,32,33,34] configuration, albeit a recent study demonstrated the effectiveness of a quadrupole-based ICP with the employment of O_2_ gas for transient broadening [23]. A recent study by Yin et al., explored the various ICP-based platforms to assess the figures of merit regarding isotope ratio measurements on single NPs and cells [25]. The conclusion for the Ag NPs (40 and 80 nm) and cells containing Ag (100 and 300 µg L^−1^) was that their ability to quasi-simultaneously (ICP-TOF) and simultaneously (MC-ICP-MS) outperformed the scanning quadruple-ICP-MS, as expected. Moreover, the MC-ICP-MS outperformed the ICP-TOF-MS such that all particle determinations were within ±5% deviation of the expected value, while the ICP-TOF-MS had 80% of the events within ±30%, although the ICP-TOF-MS ultimately offers the benefit of being able to monitor all isotopes of interest (e.g., ^6^Li–^242^Pu).

**Figure 5:**
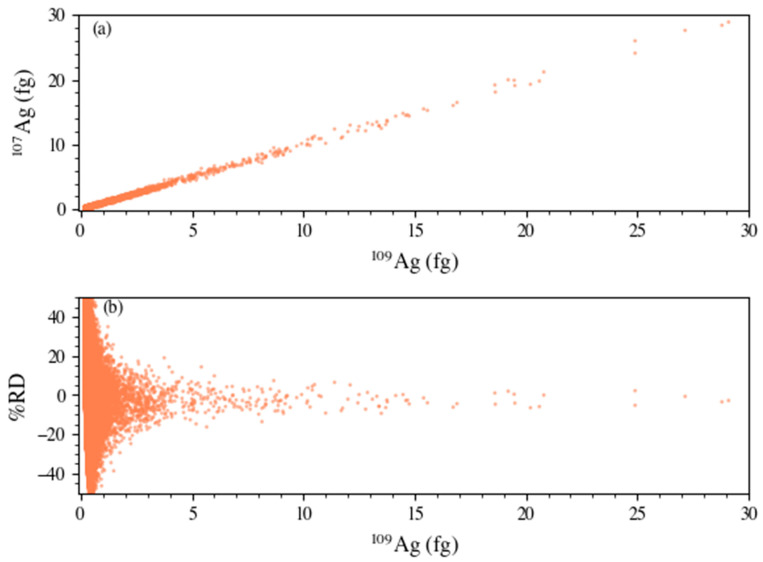
Isotopic analysis of the ^107^Ag and ^109^Ag isotopes of the Au/Ag NPs represented as a mass plot (**a**) and a percent relative difference plot with respect to mass (**b**).

From the Au/Ag NPs, the ^107^Ag/^109^Ag isotope ratio was determined. For this determination, a direct mass bias correction was applied to the NPs based on the daily analysis of the Ag ionic standard. The isotopic data presented from the entirety of the study (5 days) are presented in Figure 5, which depicts the analysis of just over 132,000 particles. When looking at the mass (fg) plot (^107^Ag versus ^109^Ag) in Figure 5a, there is a strong positive and linear correlation of the data (r = 0.988), similar to what was determined by Yin et al., when comparing the ICP-TOF-MS to other ICP-based platforms [25]. Figure 5b provides an insight to the accuracy of the measurement with respect to the mass of the characterized particles. It should be noted that each 60 nm core-shell particle should contain 1 fg of Ag (0.52 and 0.48 fg for ^107^Ag and ^109^Ag, respectively). This is a significantly smaller mass than has previously been published regarding this measurement. Similar trends have been identified previously [23,33], primarily due to Poisson counting statistics such that accurate isotope ratio determination is often hindered when analyzing low signal particles. To summarize, 80.8% of the detection population was within ±30% RD, 61.6% was within ±20% RD, and 33.7% was within ±10% RD. When considering this as a function of mass, at levels > 1.8 fg and >9.2 fg, most of the respective population (≥99%) was within ±20 and 10% RD, respectively. Based on the deviation of low signal events and their impact on isotope ratio, a threshold equal to that of the largest daily LOD (0.19 fg ^109^Ag) was applied for the final isotope ratio calculations.

**Figure 6:**
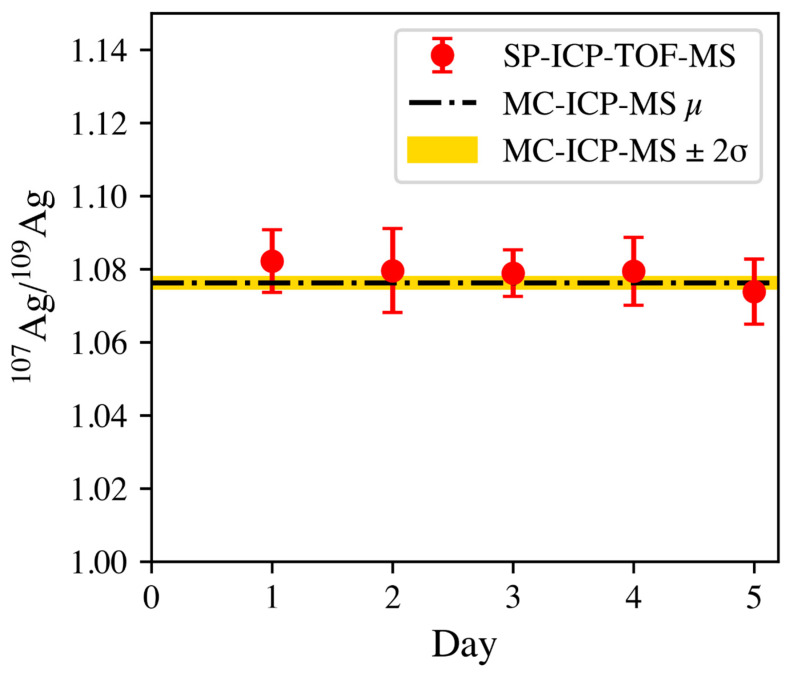
Depiction of the ^107^Ag/^109^Ag isotope ratio, determined by SP-ICP-TOF-MS, over the course of 5 days with respect to the determined value via bulk digestion-MC-ICP-MS (dashed line).

The day-to-day accuracy and precision of the isotopic determination of the Ag component within the Au/Ag NPs are presented in Figure 6. It is evident that the SP-ICP-TOF-MS method was accurate at determining this ratio when compared to the bulk digestion-based MC-ICP-MS measurement. The average ratio over the course of the days was determined to be 1.0788 ± 0.0030 (2σ) with respect to the MC-ICP-MS determination of 1.0763 ± 0.0014 (2σ). The % of RD was determined to be 0.23% over the course of the measurements.

## 4. Conclusions

Herein, the analysis of 50 nm Au, 100 nm Au, and 60 nm Au/Ag core-shell NPs employing an automated introduction system with ICP-TOF-MS was presented. This methodology includes sample mixing, precise injection, and efficient NP characterization. Ultimately, the sample was reproducibly delivered to the ICP-TOF-MS with an average %RSDs of 3.4% in-run and 9.5% day-to-day for the total number of detected particles per injection. Regarding the characterization of the NPs, all particle sizes were determined well within the certificate value with the day-to-day reproducibility of <1.03% RSD. The isotopic determination of the Ag shell of the 60 nm Au/Ag core-shell particle was also presented and had a strong linear correlation amongst the ^107^Ag/^109^Ag isotopic plot. The determined isotopic values of the 100,000+ particles were in excellent agreement (0.23% RD) with the comparator value from bulk digestion-MC-ICP-MS measurement. This methodology, automated sample introduction SP-ICP-TOF-MS, demonstrated its robustness and effectiveness regarding high in-run and day-to-day precision for NP characterization. This analytical technique lends itself to readily being applied to more routine applications where high-throughput particle characterization is warranted.

## Figures and Tables

**Table 1:** Relevant characteristics of the nanoparticle samples.

Characteristic	50 nm Au	100 nm Au	60 nm Ag/Au Core Shells
Diameter (nm)	51.0 ± 1.9	102.2 ± 4.2	59 ± 6 (total)
			Au = 30 ± 3 Ag = 15 (calculated)
Surface Area (m^2^g^−1^)	6.1	3.0	8.5
Mass Concentration (g L^−1^)	0.053	0.053	0.8 (Ag); 0.24 (Au)
Particle Concentration (particles mL^−1^)	3.9 × 10^10^	4.9 × 10^9^	8 × 10^11^
Working Particle Concentration (particles mL^−1^)	3.9 × 10^4^	4.9 × 10^4^	8 × 10^4^

**Table 2:** Autosampler sample introduction procedure.

Step	Action	Volume (µL)	Speed (µL min^−1^)
1	Probe descends into sample vial	–	–
2–5	Sample mixing	–	3000
6	Fill sample loop	150	2000
7	Probe ascends	–	–
8	Probe moves to rinse	–	–
9	Probe descends into rinse	–	–
10	Dispense from sample loop to ICP-MS	100	10
11	Fill sample loop from rinse	1000	25,000
12	Probe moves to waste	–	–
13	Dispense	1000	25,000
14	Probe moves to rinse	–	–
15	Fill sample loop from rinse	1000	25,000
16	Probe moves to waste	–	–
17	Dispense	1000	25,000

**Table 3:** Single Particle (SP)-ICP-MS example sequence with respective integration (ms) and acquisition (s) time.

Sample	Integration Time (ms)	Acquisition Time (s)
DI Water (×3)	100	60
Au standards (1, 5, 10 ng mL^−1^)	100	60
DI Water	100	60
Ag standards (1, 5, 10 ng mL^−1^)	100	60
DI Water	100	60
50 nm Au NPs (×11)	2	450
DI Water	100	60
60 nm Au/Ag core-shell NPs (×11)	2	450
DI Water	100	60
100 nm Au NPs (×11)	2	450
DI Water	100	60

## Data Availability

Data is available upon reasonable request.
